# Delayed complications from polyacrylamide gel breast fillers: a case report

**DOI:** 10.1093/jscr/rjae095

**Published:** 2024-02-22

**Authors:** Patrick Tang, Suat L Ng

**Affiliations:** Department of Surgery, Austin Health, 145 Studley Road, Heidelberg, 3084, Victoria, Australia; Department of Surgery, Austin Health, 145 Studley Road, Heidelberg, 3084, Victoria, Australia

**Keywords:** breast surgery, mammoplasty, polyacrylamide gel

## Abstract

In the late nineties, polyacrylamide gel (PAAG) gained popularity in China as a soft tissue filler for breast augmentation and contouring, but was banned 10 years later due to the increasing incidence of complications. We report a case of PAAG complication that occurred 20 years after the initial injection, where the patient had significant unilateral breast swelling and an intracapsular lesion. Surgical removal of the breast filler and immediate breast reconstruction was successfully performed, and histology confirmed a benign breast lesion. These findings highlight the importance of clinical awareness of PAAG breast filler complications.

## Introduction

Polyacrylamide gel (PAAG) is a biodegradable hydrogel that was introduced in 1997 as a soft tissue filler for breast augmentation and contouring. It gained significant popularity in China until it was banned in 2006 due to safety concerns and an increasing amount of complications [1]. It is estimated that ˃500 000 people received PAAG injections during this period [1]. Previous case series have reported that an average time for complications to occur after PAAG injection is 39–61 months [2, 3]. We report a case of PAAG complication much later, 20 years after the initial injection.

## Case report

A 48-year-old postmenopausal woman presented with a 4-month history of right breast swelling and pain. The patient had PAAG breast fillers injected in both breasts two decades ago. The breast swelling was not associated with any rash, nipple changes, discharge, or bone pain. She has no family history of breast or ovarian cancer, and was not on hormonal replacement therapy. Her past medical history includes immunoglobulin A (IgA) nephropathy, for which she has required peritoneal dialysis for 2 years and is now transitioning to hemodialysis.

On examination, the right breast was significantly larger and firmer compared to the left breast. Both breasts had minimal native breast tissue and had well circumscribed breast fillers underneath. There were no skin changes or nipple changes on the right breast and no palpable axillary lymphadenopathy. There were also no surgical scars on either breast.

An ultrasound of bilateral breasts, undertaken 2 months prior, found no solid or cystic masses in both breast parenchyma. However, it showed diffuse heterogeneous content in both breast implants and a keyhole sign within the right implant, suggestive of an intracapsular rupture. A mammogram was not performed as the patient declined due to her symptoms.

**Figure 1:**
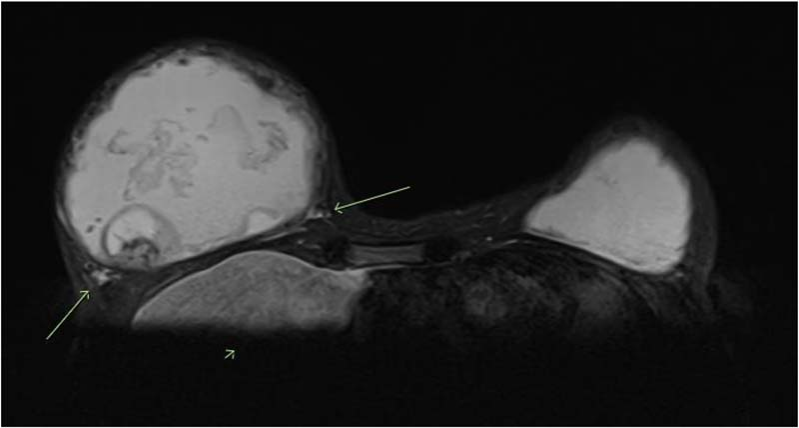
T2 weighted MRI of bilateral breasts—showing irregular borders of the fibrous capsule, heterogeneous material within the capsule, an intracapsular lesion, and extracapsular signal, suggestive of intracapsular and extracapsular breast implant rupture.

A magnetic resonance imaging (MRI) scan of the breasts showed a retroglandular breast implant/injectable filler measuring 1015 cc in the right breast. There were irregular margins and some signal external to the fibrous capsule, suggestive of an intracapsular and extracapsular implant rupture. Within the right breast filler, there was heterogeneous signal and a focal ovoid heterogeneous component with internal vascularity measuring 4.3 × 3.4 cm, at 8 o’clock 10 cm from the nipple. This was reported to be either fat necrosis or a breast implant-related neoplasm. There were no suspicious lesions in the right breast parenchyma and no axillary lymphadenopathy. The left breast also showed a retroglandular breast implant/injectable filler measuring 583 cc, and no suspicious lesions within the filler or the breast parenchyma (Fig. 1).

**Figure 2:**
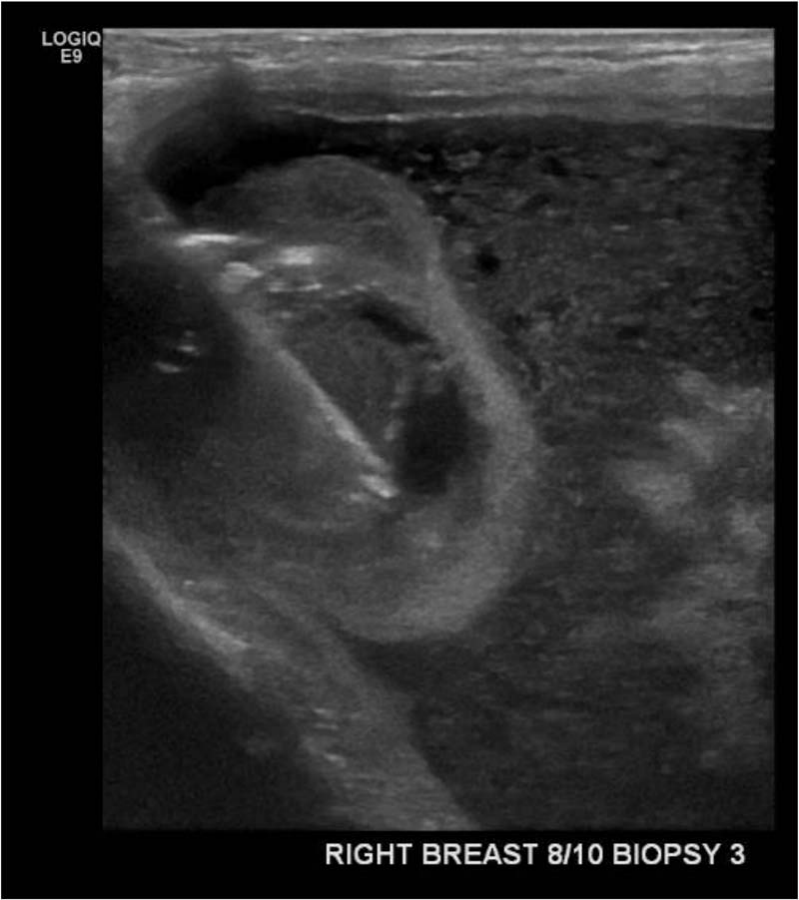
Ultrasound-guided core biopsy of the right intracapsular breast lesion.

An ultrasound-guided core biopsy of the right breast intracapscular lesion was performed (Fig. 2). The histology showed mostly fibrinous material with a small amount of exogenous foreign material, with no evidence of granulomatous inflammation or malignancy.

**Figure 3:**
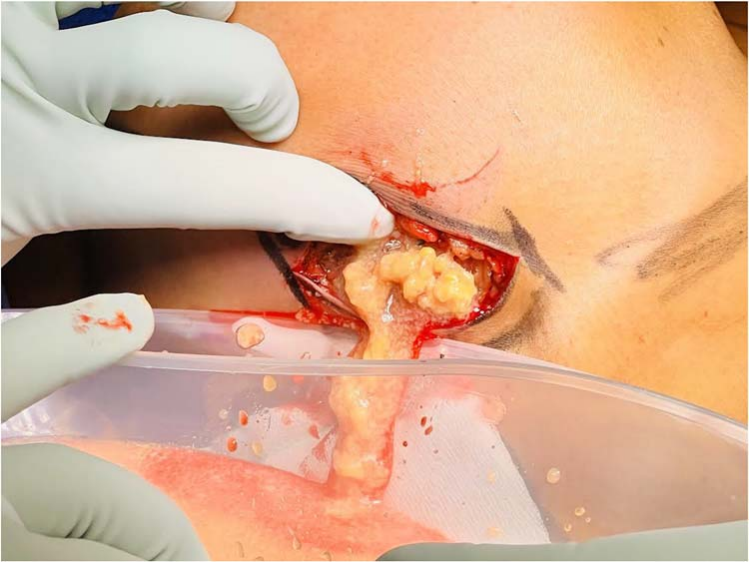
PAAG breast filler material being expressed out of an inframammary fold incision of the right breast.

**Figure 4:**
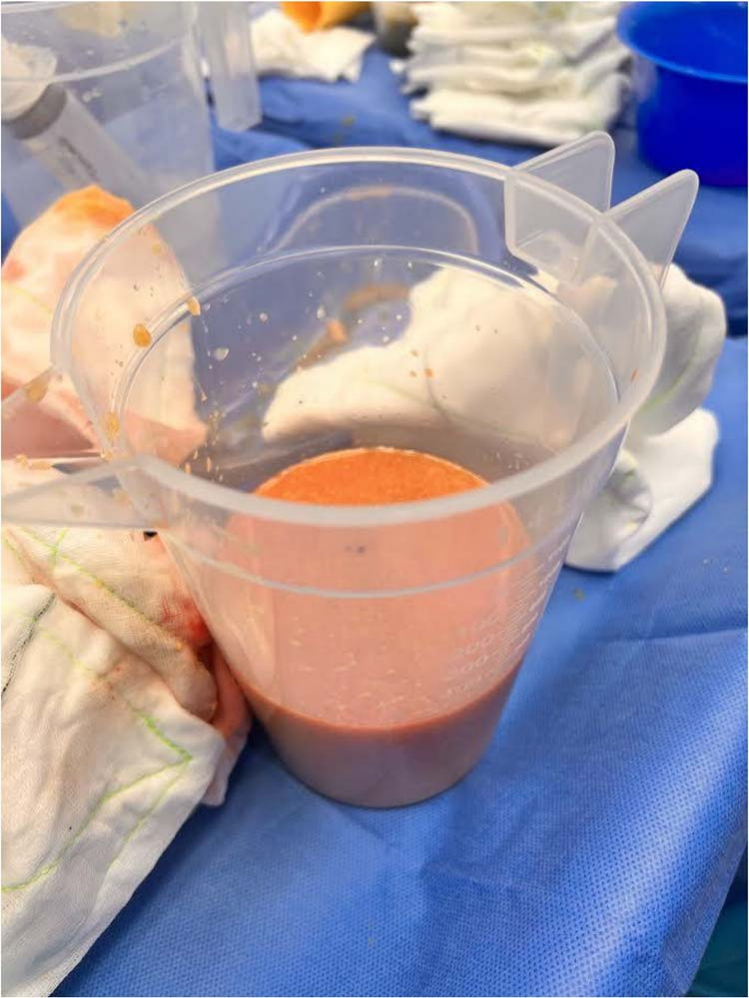
Evacuated PAAG breast filler from patient’s right breast.

The patient proceeded to have the right breast filler removed. An inframammary fold incision was made and the filler material was discharged at high pressure (Figs 3 and 4). The contents had the appearance of liquified fat. The cavity was washed out and the intracapsular lesion was excised, along with a complete capsulectomy. Given that the core biopsy was benign, breast reconstruction was immediately performed.

Postoperatively, the patient has no further issues with the right breast. The final histology of the intracapsular lesion showed fibrin only, with no evidence of malignant cells. Histology of the capsule was also benign, showing a fibrous capsule with foreign material and multinucleate giant cell reaction. Additional investigations were undertaken, including cell cytometry, tuberculosis (TB) serology, microscopy, culture and sensitivities, and these did not return any positive findings. The patient now intends to have the left breast filler removed in the near future.

## Discussion

PAAGs used as injectable breast fillers have been associated with numerous complications, which led to its eventual ban by the Chinese government. These complications include pain; swelling, infections, granulomatous tissue reactions and abscesses; filler migration; breast cancer, usually infiltrating ductal carcinomas; and nodule formation. Complications are estimated to occur in 12.38% of PAAG injections to the breast [4]. Our case report highlights that complications can arise much later than the average time, such as two decades after the initial injection. With Chinese migration to Australia over recent years, we are now seeing more complications related to PAAGs reported in Australia [5, 6].

PAAG has generally been injected in two main ways. It has been wholly injected in the retromammary space in a single position to achieve both volume and augmentation effect. It has also been injected in multiple locations over the entire breast, which is the traditional filler technique. Radiographic imaging, especially MRI, is most useful in diagnosis and for pre-operative planning, as it enables both formal assessment of the exact location(s) of the filler and accurate measurement of the volume of the injected gel [7]. Standard breast imaging, such as ultrasound or mammography, can also be useful to evaluate for any suspicious breast lesions [3, 7]. PAAG fillers can be mistaken for intracapsular and extracapsular breast implant rupture due to similar radiological features, in particular having a teardrop sign and extracapsular fluid on MRI, like our patient [8, 9]. It is important to note previous PAAG injections as a key history to other clinicians involved in these cases to ensure proper diagnosis and management, given the rarity of these cases in Western countries.

Surgical removal of the PAAG filler is required in managing PAAG complications. This is usually done via a peri-areolar or inframammary fold incision. The fibrous capsule should be removed and a thorough irrigation of the wound should be performed to remove as much PAAG as possible. Immediate breast reconstruction is usually performed after PAAG removal, due to the obvious breast deformity that would be present, which can heavily impact on patient’s quality of life [2–4]. If there is concern about malignancy or infection, staged reconstruction may be an alternative option. For follow-up, previous studies have also recommended a 6-month postoperative breast ultrasound to assess for any residual PAAG [2].

This case is particularly notable given the extended period between the initial injections of the PAAG breast fillers and their complications. Given the rarity of these cases, imaging findings can be confused with breast implant ruptures, as with our patient. Despite previous reports of breast cancers secondary to PAAG fillers, our patient fortunately had benign histologic features of her intracapsular lesion. Ongoing follow-up will be required to ensure no recurrence of complications of the right breast or new complications of the left breast.

## Conclusion

Our case demonstrates that the potential risk of PAAG-associated complications is still ever-present, and that clinical awareness is necessary for appropriate diagnosis and management. This is especially because there will likely be more cases presenting, with increasing migration from Asian countries to Western countries. Further research, such as in the form of larger prospective cohort studies, is required to determine standardized workup, management and follow-up of these complications.
